# Finite Element Analysis (FEA) of a Premaxillary Device: A New Type of Subperiosteal Implant to Treat Severe Atrophy of the Maxilla

**DOI:** 10.3390/biomimetics8040336

**Published:** 2023-07-31

**Authors:** Alessandro Cipollina, Mario Ceddia, Natalia Di Pietro, Francesco Inchingolo, Margherita Tumedei, Tea Romasco, Adriano Piattelli, Alessandro Specchiulli, Bartolomeo Trentadue

**Affiliations:** 1 Independent Researcher, 92019 Sciacca, Italy; alexandros1960@libero.it; 2 Department of Mechanics, Mathematics and Management, Politecnico di Bari University, 70125 Bari, Italy; marioceddia1998@gmail.com (M.C.); bartolomeo.trentadue@poliba.it (B.T.); 3 Department of Medical, Oral and Biotechnological Sciences, “G. d’Annunzio” University of Chieti-Pescara, 66100 Chieti, Italy; tea.romasco@unich.it (T.R.); alessandrospecchiulli@alice.it (A.S.); 4 Center for Advanced Studies and Technologies (CAST), “G. d’Annunzio” University of Chieti-Pescara, 66100 Chieti, Italy; 5 Department of Interdisciplinary Medicine, University of Bari “Aldo Moro”, 70124 Bari, Italy; francesco.inchingolo@uniba.it; 6 Department of Medical, Surgical and Dental Sciences, University of Milan, 20122 Milan, Italy; margherita.tumedei@unimi.it; 7 School of Dentistry, Saint Camillus International University of Health and Medical Sciences, 00131 Rome, Italy; apiattelli51@gmail.com; 8 Facultad de Medicina, UCAM Universidad Católica San Antonio de Murcia, 30107 Murcia, Spain

**Keywords:** edentulism, finite element analysis (FEA), maxilla atrophy, maxillary rehabilitation, sinus augmentation, premaxillary device, pterygoid implants, zygomatic implants

## Abstract

Extreme atrophy of the maxilla still poses challenges for clinicians. Some of the techniques used to address this issue can be complex, risky, expensive, and time consuming, often requiring skilled surgeons. While many commonly used techniques have achieved very high success rates, complications may arise in certain cases. In this context, the premaxillary device (PD) technique offers a simpler approach to reconstruct severely atrophic maxillae, aiming to avoid more complicated and risky surgical procedures. Finite element analysis (FEA) enables the evaluation of different aspects of dental implant biomechanics. Our results demonstrated that using a PD allows for an optimal distribution of stresses on the basal bone, avoiding tension peaks that can lead to bone resorption or implant failure. ANSYS^®^ was used to perform localized finite element analysis (FEA), enabling a more precise examination of the peri-crestal area and the PD through an accurate mesh element reconstruction, which facilitated the mathematical solution of FEA. The most favorable biomechanical behavior was observed for materials such as titanium alloys, which helped to reduce stress levels on bone, implants, screws, and abutments. Additionally, stress values remained within the limits of basal bone and titanium alloy strengths. In conclusion, from a biomechanical point of view, PDs appear to be viable alternatives for rehabilitating severe atrophic maxillae.

## 1. Introduction

People with edentulism may experience feelings of disability for different reasons, such as reduced chewing efficiency, speech difficulties, and poor facial aesthetics [1]. Dental implantology has successfully addressed the challenges of anatomical consequences and is now recognized as a successful option for rehabilitation [2,3]. In particular, osseointegration is one of the fundamental aspects of implantology, which was described for the first time by Brånemark et al. in 1985 [4], leading to a revolution in the dentistry field. Osseointegration is the phenomenon whereby the dental implant fully integrates, ensuring its long-term stability when there is sufficient quantity and quality of bone. However, the amount of bone is often insufficient, particularly in cases of edentulism, which results in a lack of bone stimulation and leads to issues, such as bone resorption and atrophy. Treating severe atrophy of the maxilla remains a significant challenge for clinicians. Severe maxillary atrophy involves a significant resorption of alveolar bone, particularly in the upper jaw, which is a crucial area for the placement of dental implants to support future dental prosthesis for chewing restoration.

Severe maxillary atrophy, particularly in the premaxilla area, is typically addressed through methods, such as large maxillary sinus lifts with bank bone insertion or autologous bone grafts [5,6]. In both cases, these methods involve long, expensive, and painful procedures, requiring a considerable amount of time to determine their success or failure. Additionally, there are no guarantees of complete healing and predictable recovery of the alveolar bone, and complications have also been reported, primarily including sinusitis. Alternatively, several surgical and reconstructive techniques have been proposed, such as inlay/onlay grafts and dental implants, guided bone regeneration (GBR), distraction osteogenesis, splitting of the alveolar ridge, Lefort I interpositional grafts, use of inclined implants, zygomatic implants, and pterygoid implants [7,8,9,10,11,12].

All of these techniques, however, can be extremely complex to perform, and they should only be carried out by highly experienced surgeons. Furthermore, they can be expensive and time-consuming. Although most of these clinical approaches have been reported to have very high success rates, complications can occur, and sometimes they can be very serious [8,10]. Among the various mentioned techniques, zygomatic implants (ZIs) offer a valid alternative to more invasive methods, providing safe, reliable, and predictable results [7,8,9,10,11]. ZIs offer several advantages over other techniques, including lower costs; apparently fewer complications; often mild, easy-to-manage, and shorter time required for prosthetic rehabilitation; and fewer prosthetic needs [7]. However, it is important to note that some complications associated with ZIs may be underestimated, and they encompass various issues, such as sinusitis, intraoral soft-tissue infection, oro-antral fistula, facial–periosteal hematoma, gingival hyperplasia, infraorbital paresthesia, penetration and perforation of the orbital cavity, prosthetic fit problems, temporary sensory nerve deficits, moderate nosebleeds, subcutaneous malar emphysema, and peri-implant soft tissue infection [8]. Pterygoid implants (PIs) have also been used successfully for treating extreme jaw atrophy [9]. These implants are generally stable and enable the anchorage of the atrophied or resorbed posterior maxilla without the need for sinus lift or bone grafts, resulting in long-term stability.

The latter is a key point in all the aforementioned techniques, and to ensure the long-term durability of an implant system, the biomechanical interaction between bone and implant plays a fundamental role. In this regard, various methods are used to assess stress around implant systems, including photoelasticity, finite element analysis (FEA), and strain measurement on the bone surface. In particular, FEA is a numerical modeling technique that was initially developed for structural analysis in mechanical, civil, and aeronautical engineering. FEA basically involves creating a numerical model that uses algorithms to analyze stresses and deformations for any type of geometry. The three-dimensional (3D) geometry is discretized into elements known as meshes, which are connected through nodes. The accuracy of the results depends on parameters such as the element size used for discretization, the type of element, and the number of elements used in the study. Recently, the FEA method has also been utilized to study the interaction between dental implants and bone, providing valuable information for clinical applications [13,14,15,16,17]. Some authors [18,19] have compared FEA studies and showed that the results, when combined with in vivo strain gauge measurements, correspond to clinical outcomes.

The aim of this study was to conduct a biomechanical evaluation of a new subperiosteal device, namely, a premaxillary device (PD), inserted in the anterior region of the maxilla. In fact, the application of FEA methodology under physiological and pathological loading conditions in the mouth is valuable for providing clinically relevant information on the failure and fatigue of the implant structure, as well as the effects of osseointegration.

## 2. Materials and Methods

### 2.1. Three-Dimensional (3D) Model

**Figure 1:**
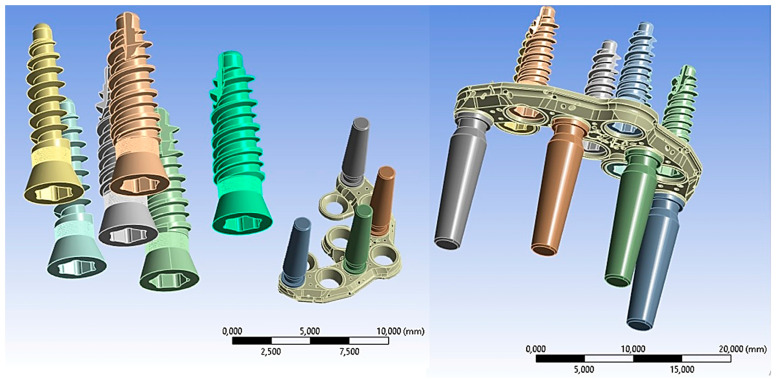
Premaxillary device (PD) components.

**Figure 2:**
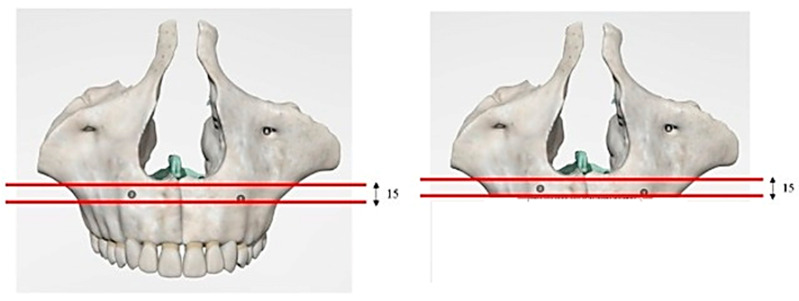
Three-dimensional (3D) model of the jaw with severe atrophy of the maxilla.

**Figure 3:**
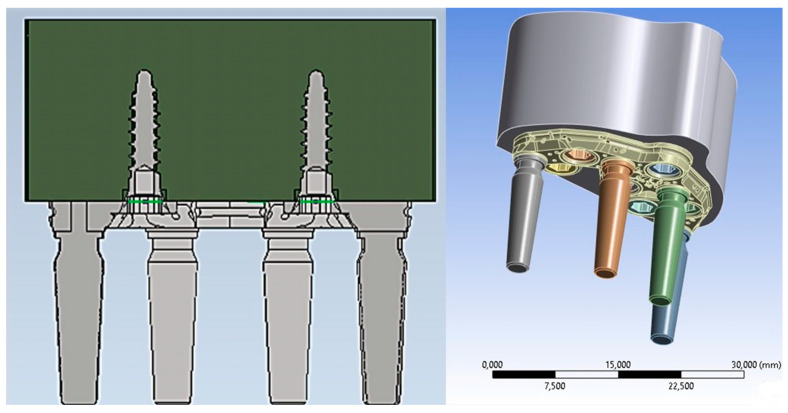
A 3D model of the PD placed on the bone model.

Computer-aided design (CAD) software (Autodesk Inventor 2023, San Francisco, CA, USA) was used to create a 3D model of the implant system, which included the PD, fixing implants, and their corresponding abutments (Aldo Corbo Research and Innovation Srl, Canicattì, Agrigento, Italy) (Figure 1). Moreover, a virtual 3D model of a completely edentulous jaw was selected from the computed tomography (CT) database of the Renato Archer Information Technology Center (CTI, Campinas, São Paulo, Brazil). Using various 3D-editing tools, the jaw was cut to simulate severe bone atrophy (Figure 2). Based on similar studies [10], an atrophy of 8–15 mm was targeted. The removed portion was later used to create the simplified model, and then the PD was placed on the bone model (Figure 3).

The conventional insertion of an implant must be supported by an adequate amount of bone. Considering that for the upper maxilla it is possible to exploit all the bone height available between the bone crest and the floor of the maxillary sinus, implants of 12 mm in length were considered, taking into account a PD thickness of about 2.5 mm.

### 2.2. Material Properties

The bone on which the PD is positioned is the basal bone, which has different mechanical characteristics from both cortical and trabecular bone. The elastic properties (elastic modulus and hardness) of the bone in contact with the implant play a fundamental role in determining the stability and success of the implant. In this regard, several publications have described the mechanical properties of cortical and spongy bone, but there are few studies in the literature regarding the characteristics of basal bone [11,20,21,22]. However, through knowledge of the density and using mathematical relationships, it was possible to determine the value of the stiffness. Clinical bone density data can be accurately detected by CT analyzed with specific programs for dentistry. The CT data assign to each voxel a number that is dependent on the average density of tissues in that specific unit volume. This number, which can be highlighted on the X-ray areas of interest (ROI: region of interest), is part of a standardized scale of densities expressed in Hounsfield Units (HU). It can take values between −1500 and +2595, and it assigns the density of water a value of 0 and that of air a value close to −1500. Bone structures on the Hounsfield scale vary between +150 and +1500. It is possible to relate the data in HU according to the Misch classification [23], as shown in Table 1.

**Figure 4:**
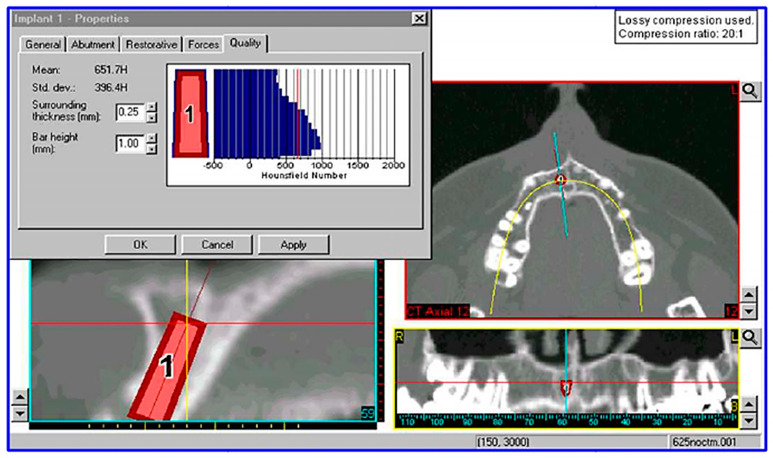
Measurement of bone density in Hounsfield Units (HU) at a virtual fixture insertion site using a computed tomography (CT) image processed with Simplant software 18.5 (Materialise HQ, Leuven, Belgium).

Based on a study conducted by other authors [24] (Figure 4), it was inferred that the HU density of the jaw is approximately 650 HU, indicating that the jaw can be classified as type D3 bone according to Misch’s classification. Therefore, the density was estimated to be 0.62 g/cm^3^.

In a study conducted by Seong et al. [11], where the elastic properties of the edentulous maxilla and mandible were evaluated, the following mechanical properties were obtained and are reported in Table 2.

For implants, abutments, and the PD, Ti6Al4V titanium alloy was used as the material, and its related mechanical properties are shown in Table 3 [12]. Titanium, employed for the production of all components, presents some issues, including the problem of stress shielding and consequent loss of implant and bone. This phenomenon is caused by its high elastic modulus (110 GPa) compared to bone (14 GPa).

For both materials, isotropic behaviors were assumed, and thus the mechanical characteristics were assumed not to change with the direction.

### 2.3. Finite Element Model (FEM)

Models previously constructed using CAD software were processed and exported to finite element software (ANSYS 2023, Workbench, Canosburg, PA, USA). The simulation hypothesis assumed linear elastic behavior and homogeneous, isotropic characteristics for the materials, as described by Young’s modulus and Poisson’s ratio in Table 2 and Table 3.

**Figure 5:**
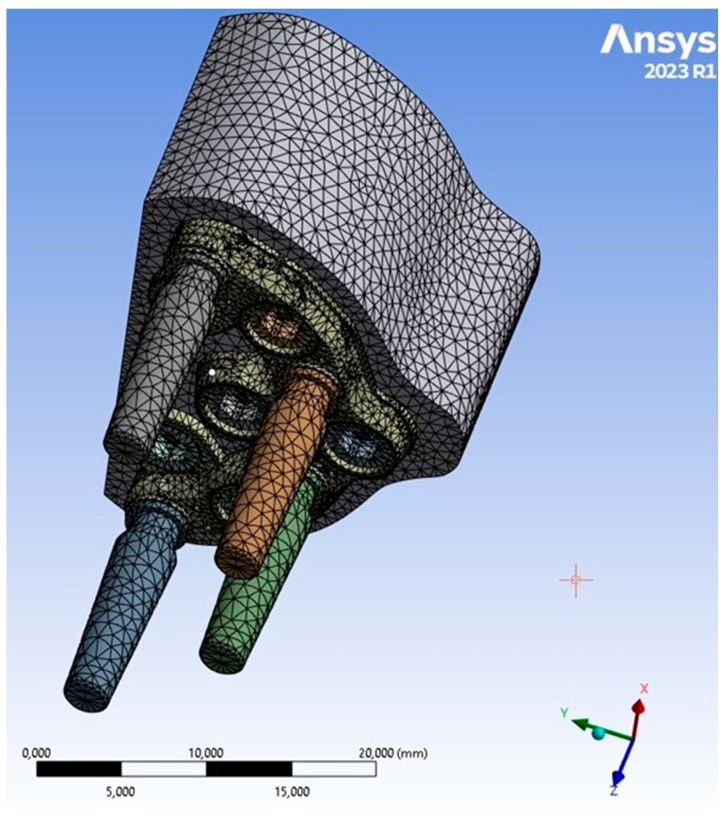
PD mesh model.

The PD was securely attached to the jaw, and abutments–implants were inserted into their corresponding housings. To achieve high computational accuracy, a mesh with a resolution of 0.5 mm was used to discretize 3D model into small elements. Four distinct types of mesh elements were used: linear tetrahedral, quadratic tetrahedral, linear hexahedral, and quadratic hexahedral. The quadratic mesh element employs a nonlinear form function that interpolates nodes using a higher-order polynomial. ANSYS recommends tetrahedral mesh elements as the preferred choice for complicated nonlinear geometries. Hence, the default option for creating the element type was selected [13]. Tetrahedral elements were applied to all structures with minimum and maximum dimensions ranging from 0.15 to 0.7 mm. Regions with higher stress levels were manually refined to gain better control over the actual stress distribution at fittings and edges. The 3D models consisted of a substantial number (2,658,021) of elements (Figure 5).

**Figure 6:**
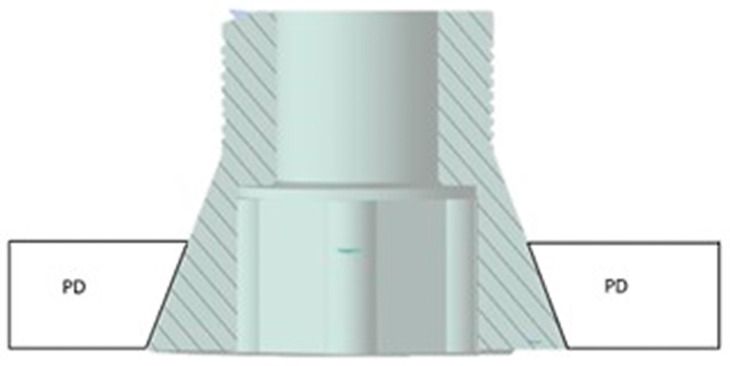
Cone–Morse connection at the screw–PD interface.

Before conducting the FEA study, it was essential to simulate the contact between the PD screw and the PD abutment. This involved assigning the corresponding contact surface and specifying contact conditions to accurately simulate the system. In particular, as regards the type of connection between the implant and the PD, as seen in Figure 1, it was evident that the taper on the hole of the PD was the same as on the screw head. Due to this similarity, we can conclude that there was a conical connection (Cone–Morse) at the screw–PD interface (Figure 6).

**Figure 7:**
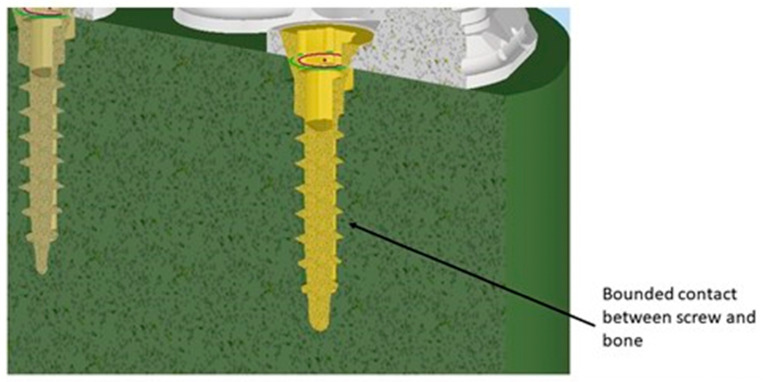
Contact between screw and bone.

Another crucial aspect relates to the issue of any relative micromovement at the bone–implant interface. It is now evident that to ensure successful bone regeneration and prevent implant failure, achieving an optimal level of osseointegration is essential, and a fundamental requirement for this is the immobility of the implant within the implant site. Therefore, to model the bone–implant contact, a fixed connection between bone and screw was employed. This choice restricted movements in all three directions (x, y, z) and their corresponding rotations, ensuring that the screw remained immobile inside the bone (Figure 7).

**Figure 8:**
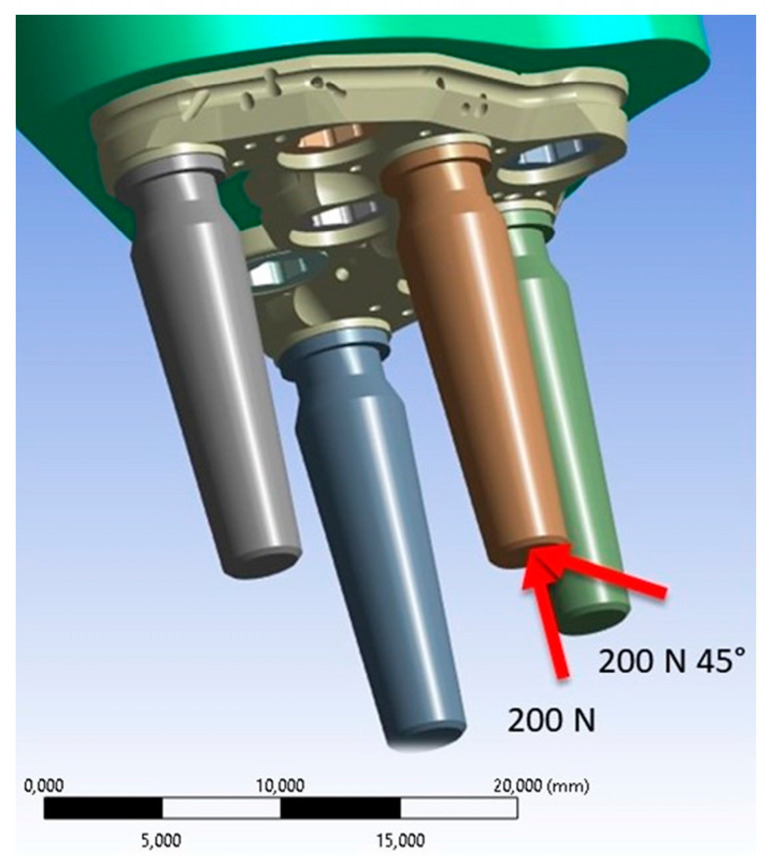
Application of vertical and oblique loads (red arrows).

The occlusal surface of the abutment was subjected to two loading conditions. The first condition involved the application of a load of 200 N in the apical direction, while the second condition applied the same load at an inclination of 45° (Figure 8). In this way, the variation of the maximum Von Mises stress with changes in inclination was highlighted [14].

## 3. Results

**Figure 9:**
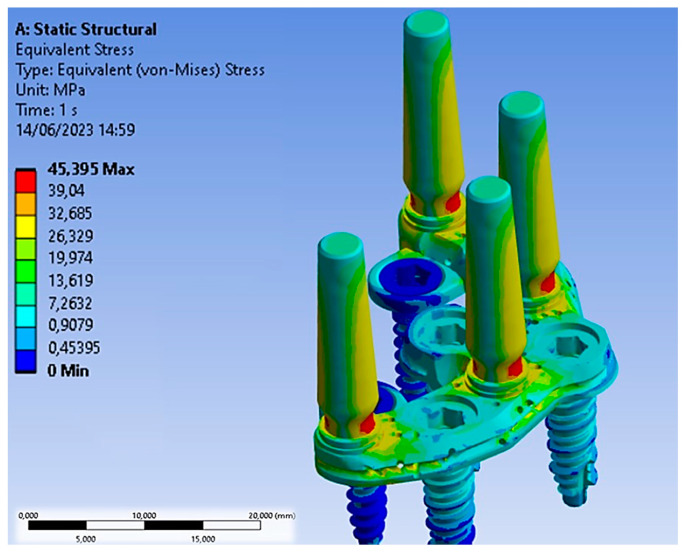
Von Mises stress distribution after the application of a load of 200 N along the apical direction.

Concerning the application of the 200 N load along the apical direction, Figure 9 shows the distribution of Von Mises stress for the entire system.

The Von Mises stress distribution was uniformly spread over the entire surface of the PD, with some alterations near the abutments, where forces were exchanged with the outside. Specifically, stress values on the PD ranged between 5 and 15 MPa. The abutments, being the components directly subjected to the load, experienced the highest stress. In particular, the conical geometry demonstrated a stress distribution decreasing from the upper area of the abutment (yellow region) from 27 MPa to approximately 13.62 MPa (green region). The narrow section at the threaded connection is the area where stresses reached values of about 34.40 MPa, making it the most critical area in the entire system due to the higher stress values recorded.

**Figure 10:**
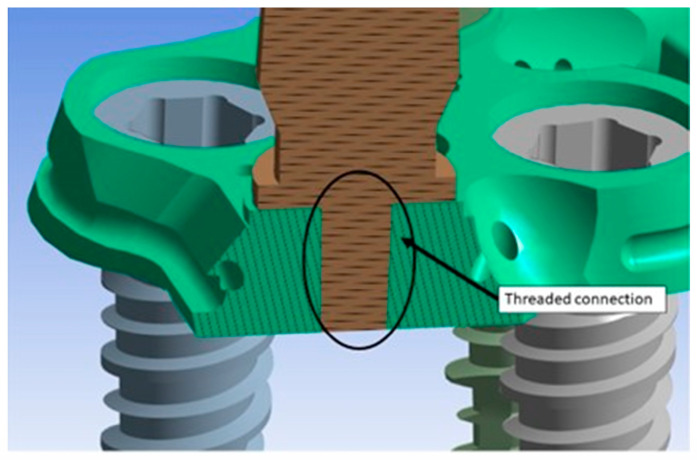
Threaded connection between the abutment and the PD.

**Figure 11:**
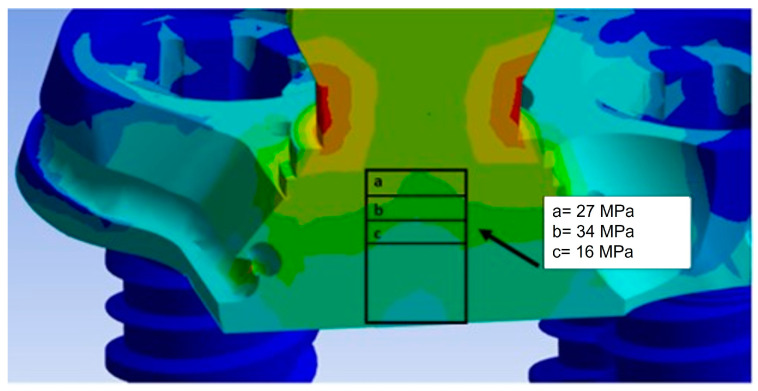
Von Mises stress distribution in the threaded connection.

The abutments were connected to the PD through a threaded connection, as shown in Figure 10. This connection involved the application of a tightening torque of 10 Ncm, which stressed the threads of the torsion and traction abutment. Since this area is the most critical, Figure 11 shows the Von Mises stress distribution between the abutment threads and the PD. It was observed that the first three threads experienced the highest stress, with values ranging between 20 MPa and 45 MPa. In future structural optimization, it may be considered to further reduce the thickness of the PD, considering that not the entire length of the abutment thread exchanges forces with the PD.

**Figure 12:**
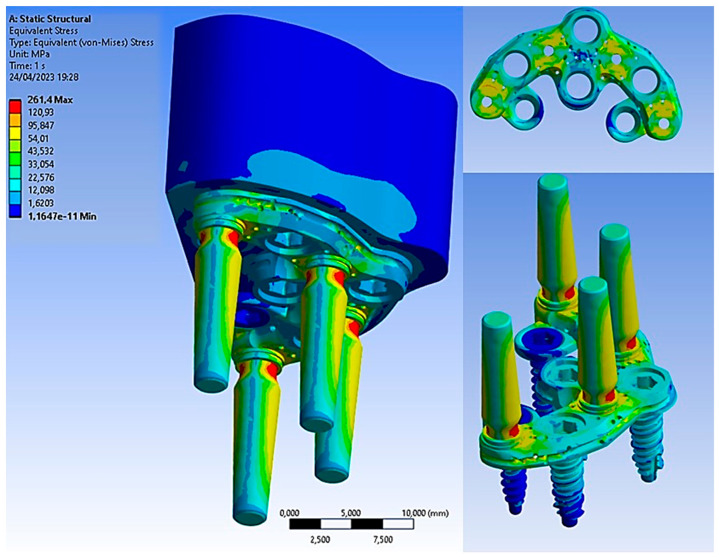
Von Mises stress on all components.

However, when the load of 200 N was applied at a 45° inclination, it resulted in increased stress on all components, including the bone, which was simulated in this configuration, as it is the most critical. The maximum Von Mises stress values are shown in Figure 12.

**Figure 13:**
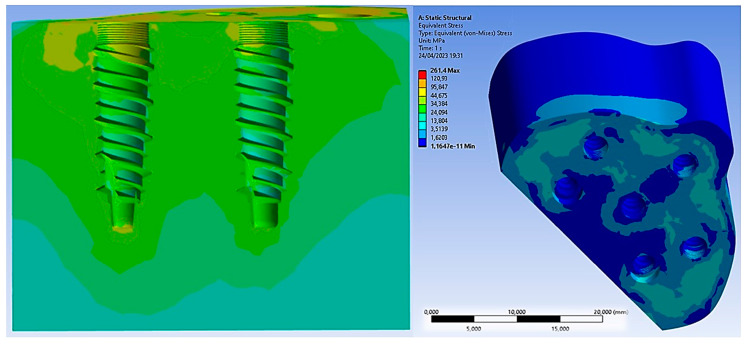
Von Mises stress on basal bone.

The basal bone experienced maximum stress concentration, mainly at the bone interface region and the PD, ranging from 3 to 15 MPa. Due to its geometry, the PD could evenly distribute Von Mises stresses. Figure 13 illustrates stress values ranging from 40 MPa to 250 MPa. These values suggest the potential for further structural improvement on the PD to reduce its thickness and limit damage to soft tissues. Notably, the yield stress of the Ti6Al4V titanium alloy was approximately 800 MPa, providing a safety factor of about 3.

As for the implants, the maximum stress was observed around the neck of the implant and between the first and third threads, reaching 22 MPa. On the other hand, the abutments were the most stressed components, experiencing 270 MPa of stress. This stress was induced by the application of the inclined load at 45°, leading to bending at the base of the abutment. Therefore, a design improvement at this area could enhance the mechanical resistance, especially considering that masticatory loads vary over time, subjecting the entire system to the phenomenon of fatigue, which can lead to failure with loads lower than static ones [15,16,17].

**Figure 14:**
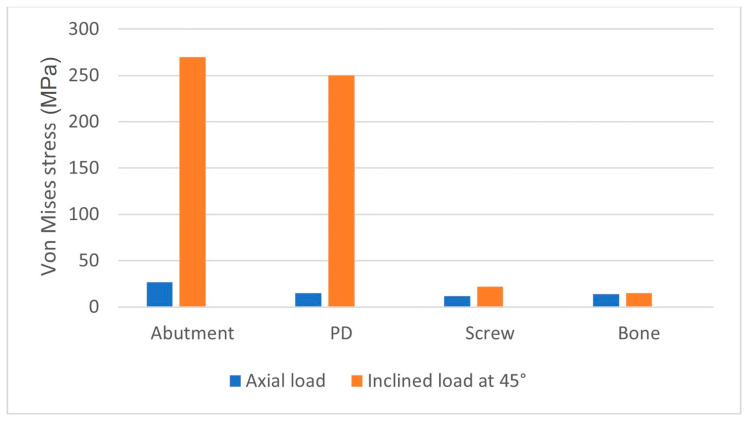
Von Mises stress on all components after the application of the load in the axial direction and at a 45° inclination.

Comparing the results with the application of the load in the axial direction and at a 45° inclination, we could deduce the results shown in Figure 14. It was observed that the stress acting on the bone was not strictly dependent on the angle of the load. However, upon closer observation of the abutment, it is evident that the stress value increased from 34.40 MPa to approximately 270 MPa. This demonstrates the critical importance of the tangential component of the load for the system.

## 4. Discussion

Maxillary sinus augmentation is a viable technique that can be used to insert implants in a maxilla with reduced height. However, this technique can lead to several complications, which can be classified into three categories: intraoperative, acute postoperative, and chronic postoperative complications. In particular, the most frequent complications include perforation of the Schneiderian membrane, intraoperative hemorrhage, injury to the infraorbital nerve, perforation of the orbital wall, implant displacement within the sinus, edema, infection of the inserted graft, flap dehiscence, and formation of a fistula [25,26,27,28,29,30,31,32,33,34,35,36].

Thus, a valid alternative could be to use the ZI procedure, which has been reported to have very high survival rates. However, even with this procedure, there are numerous studies mentioning that the use of ZIs is not without complications. The insertion of ZIs represents a major surgical procedure that should be performed under general anesthesia by properly trained surgeons. The learning curve for ZIs can be very long, and the brain and orbit may be affected by the procedure [37]. In recent years, static and dynamic navigation techniques have been utilized [38]. In some cases, when implants are inserted in a more palatal position, a more complicated prosthetic restoration may be necessary [37]. Additionally, the removal of a failed implant could be a more complex procedure. Complications associated with ZIs include sinusitis, reported in 3.9% of cases, and failure to achieve implant osseointegration, occurring in 2.44% of cases [38]. In another review, the complication rate was 7.2% when using an intrasinusal technique and 1.8% with an extrasinusal technique [27]. A 4.9% prosthetic complication rate was reported, with a 0.69% implant failure rate. In a clinical study on 141 implants inserted in 45 patients, an overall complication rate of 5.67% was reported [39], whereas in another clinical study on 182 ZIs in 82 patients, a low complication rate was reported, with sinusitis at a 10% rate and peri-implant hyperplasia at a 0.8% rate. Other reported complications include oro-antral fistula, foreign body reaction, difficulties in maintaining proper oral hygiene in the posterior palatal region, transversal mobility, paresthesia, bruising, laceration of the lips, injury to the orbit and periorbital hematoma, cranial penetration, temporary deficits of some sensory nerves, soft tissue hypertrophy, abutment and prosthetic screw loosening, mucositis, and prothesis fracture [39,40,41]. In a recent systematic review, the authors concluded that ZIs are not recommended as a first therapeutic option [36].

On the other hand, PIs have shown a success rate of 97.05% after one year [10], and a systematic review reported the same cumulative survival rate of PIs over a 10-year period [34]. In another systematic review on 1983 PIs in 634 patients, a mean survival rate of 94.87% was reported [42]. Bidra et al. [9] reported a 95.5% cumulative survival rate of PIs after 6 years. Recently, a FEA study on PIs was also published [43].

Subperiosteal implants were initially introduced in Sweden during the 1940s and widely used in the 1950s and 1960s [44]. However, they had a high percentage of complications and failure. The construction technique of this type of implant was extremely complex, obtaining a perfect adaptation to the underlying bone was very difficult, and the surgical technique was time-consuming. Moreover, these implants tended to be large, requiring substantial flaps for positioning on the bone bed. Two surgeries were necessary for bone impression and implant positioning, resulting in high biological cost. The materials used were chrome–cobalt and vitallium [45]. Stvrecky et al. [46], in a 15-year retrospective study, reported a 5–10-year survival rate of 58.3% of cases. In recent years, the reintroduction of subperiosteal implants has been facilitated by new digital technologies and metals; 3D metal printing has improved implant manufacturing accuracy, and the use of different metals, such as titanium, has resulted in smaller implant structures [47,48,49]. Modern subperiosteal implants offer advantages, such as reduced treatment period, cost reduction, and avoidance of complex and risky surgical procedures [50]. Moreover, no resorption of the underlying bone, mobility, or implant fracture, as well as a 95% survival rate, have been reported [50]. However, several studies in the literature have involved small patient populations with relatively short follow-up periods. Reported complications include swelling, edema, pain, and implant exposure [48]. Generally, patients have positively responded to the treatment, experiencing enhanced comfort, chewing capabilities, and prosthetic restoration stability [49]. These results are probably related to the use of new digital technologies, which allow for an extremely precise and close apposition of the implant structure to the underlying bone. In studies involving ten patients with a one-year follow-up, a 100% implant survival rate was reported, with 10% early complications and 20% late complications [50]. In 16 patients with the same follow-up period, no complications or implant losses were reported [51].

The stresses caused by the prosthesis during chewing cannot be directly measured in vivo. Experimental methods, such as strain gauges using electrical strain gauges and photoelasticity, have been utilized. However, each method has its limitations. For instance, strain gauges have the disadvantage of being limited by the area where they are applied, which may not include the specific area of interest. On the other hand, photoelasticity allows one to identify stress gradients over the entire structure, but it can be challenging to recreate a reflective model, especially for complex structures [52,53,54].

The FEA method has proven to be a valuable tool for estimating stress and strain in this innovative implant system. One of the strengths of FEA lies in its physical similarity between real in vivo results and numerical results. However, simplifying the geometry too much can lead to inconsistent results [52,53,54,55]. In order to obtain consistent results with FEA, the complete geometry of the implant and surrounding bone needs to be modeled, along with material properties, loading constraints, and conditions, and mesh-convergence tests must be conducted. The main advantages of the FEA methodology include its non-invasive nature, allowing for static and dynamic tests to be performed. Additionally, the study can be conducted multiple times, and there is no need to sacrifice animals, making it ethically beneficial.

However, this methodology also has drawbacks that primarily relate to the familiarity with the software, the fact that the results are influenced by configuration parameters, and the necessity of comprehensive knowledge about the behavior of the analyzed components. Another crucial aspect to consider is the presentation of results, which are provided through Von Mises stress analysis. To validate these FEA results effectively, it is best to simultaneously conduct in vivo experimental studies [52]. In fact, FEA is a numerical investigation method that cannot realistically simulate the behavior of tissues or fully represent the complexity of the biological field, and it can suffer from possible numerical errors. In fact, consistent with other FEA studies [56,57,58], all materials were considered homogeneous, isotropic, and linearly elastic, and 100% osseointegration was assumed between the bone and the implants, although such assumptions are not realistic in clinical practice.

Overall, although there are multiple advantages to use this method in reproducing approximate and predictive results, numerous randomized clinical trials on this topic must be performed to obtain reliable and definitive results. In addition, other studies are needed to simulate all treatment alternatives for atrophic jaws to include dynamic forces reproducing chewing, to consider the anisotropic and regenerative properties of natural bone, or simply to test other implant designs and prosthetic connections, such as in previous works [59,60].

## 5. Conclusions

In this work, the stress distribution on the entire PD device, involving its abutment and implant components, was numerically investigated by means of a 3D linear, elastic, and static FEA study.

On the basis of the previous results, the following conclusions can be made:

- The PD treatment concept demonstrated highly favorable biomechanical behavior and can be regarded as a viable alternative for rehabilitating severe atrophic maxilla;
- The use of highly rigid materials, such as titanium alloys, exhibited the most favorable biomechanical behavior and resulted in reduced stress levels for bone, implants, screws, and abutments;
- Stress values did not exceed the bone strength limits of the basal bone and titanium alloy;
- The application of inclined load increased stress in all areas.

However, due to the difficulties involved, such as the simulation of the entire natural oral environment and the numerical nature of the investigation methodology, researchers often use the finite element method to simulate implants and bones [56,57,58].

In conclusion it can be said that the FEA method can effectively be used to study the biomechanical behavior of implants and other devices, such as PDs, with a good level of accuracy.

## Figures and Tables

**Table 1:** Correspondence between Hounsfield Units (HU) and bone density classifications.

Hounsfield Units (HU)	Bone Density Classification
HU > 1250	Misch D1
850 < HU < 1250	Misch D2
350 < HU < 850	Misch D3
150 < HU < 350	Misch D4
HU < 150	Misch D5

**Table 2:** Mechanical properties related to the basal bone.

**Basal Bone**	**Young’s Modulus (GPa)**	**Poisson’s Ratio**
	14.5	0.3

**Table 3:** Mechanical properties of titanium alloy used for the PD.

**Titanium Alloy** **(Ti6Al4V)**	**Young’s Modulus (GPa)**	**Poisson’s Ratio**
	110	0.35

## Data Availability

All experimental data to support the findings of this study are available from the corresponding author upon request.
